# Vascular and plaque imaging with ultrasmall superparamagnetic particles of iron oxide

**DOI:** 10.1186/s12968-015-0183-4

**Published:** 2015-09-18

**Authors:** Shirjel R. Alam, Colin Stirrat, Jennifer Richards, Saeed Mirsadraee, Scott I. K. Semple, George Tse, Peter Henriksen, David E. Newby

**Affiliations:** Centre for Cardiovascular Science, The University of Edinburgh, The Chancellor’s Building, Little France Crescent, Edinburgh, EH16 5SA UK; Department of Cardiology, Royal Infirmary of Edinburgh, Edinburgh, EH16 5SA UK; Clinical Research Imaging Centre, University of Edinburgh, Edinburgh, EH16 5SA UK; Department of Radiology, Royal Infirmary of Edinburgh, Edinburgh, EH16 5SA UK; MRC Centre for Inflammation Research, The University of Edinburgh, Edinburgh, EH16 5SA UK

**Keywords:** USPIO, Nanoparticles, CMR, Cardiovascular imaging, Inflammation, Macrophage

## Abstract

Cardiovascular Magnetic Resonance (CMR) has become a primary tool for non-invasive assessment of cardiovascular anatomy, pathology and function. Existing contrast agents have been utilised for the identification of infarction, fibrosis, perfusion deficits and for angiography. Novel ultrasmall superparamagnetic particles of iron oxide (USPIO) contrast agents that are taken up by inflammatory cells can detect cellular inflammation non-invasively using CMR, potentially aiding the diagnosis of inflammatory medical conditions, guiding their treatment and giving insight into their pathophysiology. In this review we describe the utilization of USPIO as a novel contrast agent in vascular disease.

## Introduction

Inflammation is central to many cardiovascular pathophysiological processes including atherosclerosis, myocardial infarction and heart failure. Macrophages are key mediators of these inflammatory pathways, initiating both destructive and reparative processes [1]. Quantification and characterization of tissue macrophage activity may therefore assist in our understanding of the pathogenesis of cardiovascular disease and help determine disease severity and prognosis, as well as providing a biomarker to assess the efficacy of established or novel therapeutic interventions.

Cardiovascular magnetic resonance (CMR) is a well-established clinical imaging modality offering excellent soft tissue contrast and spatial resolution, whilst avoiding ionizing radiation. Standard gadolinium-based contrast agents are paramagnetic and are infused into the blood pool with variable organ extraction rates, although subsequent extravasation and redistribution can be used to identify the interstitial and extracellular spaces. Gadolinium is commonly used as an CMR contrast agent after acute myocardial infarction (MI) to identify areas of tissue infarction and fibrosis [2, 3]. Tissue oedema and rupture of cell membranes with consequent diffusion of gadolinium into the inter- and intra-cellular spaces [2] results in a “delayed gadolinium enhancement” effect in infarcted regions. Recent interest has turned to novel agents that provide additional structural and functional cellular information. Such ‘smart’ contrast agents include iron oxide nanoparticles.

### Iron oxide nano-particles

Particles of iron oxide are divided into classes based on their size (Table 1). In this review, we will focus on ultrasmall superparamagnetic particles of iron oxide (USPIOs) that consist of nanoparticles with a diameter of <50 nm and include ferumoxtran-10 (Sinerem, Guerbet) and ferumoxytol (Rienso, Takeda; Feraheme, AMAG Pharmaceuticals). Although Rienso had been authorised for use in European Union, Takeda since has withdrawn it. However Feraheme is clinically available in the United States for the treatment of iron deficiency anemia in adult patients with chronic kidney disease (CKD).

**Table 1:** Iron oxide nanoparticle preparations

Particle	Size (Diameter)	Plasma half-life (h)	Application
Microparticles of iron oxide (MPIOs)	1–6 μm [53]	1–2 min	Readily endocytosed and detected with CMR [53]. Need immediate scan following infusion.
			Can be combined with ligands for cellular targets allowing molecular imaging [54].
			Large size means they remain in the blood pool and are suitable for endovascular imaging t [55].
Superparamagnetic particles of iron oxide (SPIOs)	65–150 nm [56]	2–3 h	Ferumoxide (Endorem, Guerbet, France) and ferucarbotran (Resovist, Bayer-Schering Pharma, Germany).
			Recognised by cells of the reticuloendothelial system. Have been used for oncological imaging including liver studies where they are taken up by Kuppfer cells in normal tumour-free liver [57].
			Mesenchymal stem cell, monocyte/macrophage labelling [58].
Ultrasmall SPIO (USPIOs)	<50 nm [59]	Ferumoxytol: 9–15 h	Ferumoxtran-10 (Sinerem, Guerbet, France) and ferumoxytol (Rienso, Takeda, United Kingdom).
		Ferumoxtran-10: 25–30 h	
Very small superparamagnetic iron oxide particles (VSOPs)	<10 nm [60]	1 h	Alternative blood pool agents with longer circulating half-life than gadolinium based agents [61, 62].
			Potential as cell tracking agents [63].

Ferumoxytol is well tolerated by patients with chronic kidney disease and iron deficiency anaemia, and had a similar overall treatment-related adverse event rate to oral iron [4]. This safety data is further supported by additional retrospective observational data from three large haemodialysis clinics in the United States involving more than 8600 patients and more than 33,300 administered doses of ferumoxytol [5, 6]. The only contraindications to use are known hypersensitivity or iron overload. Therefore there is little to limit widespread clinical use as an imaging agent.

**Fig. 1:**
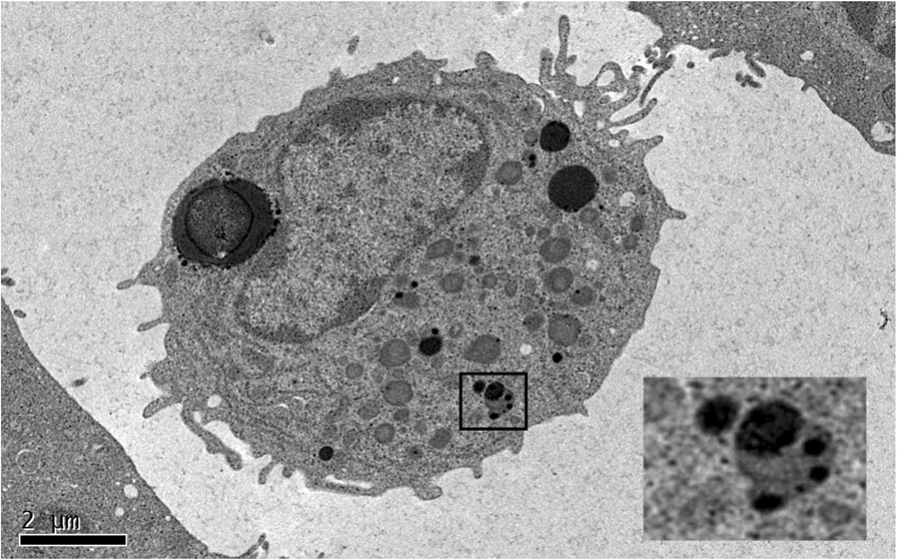
Murine blood monocyte in peripheral circulation 48 h after infusion of USPIO. Inlay (bottom right – magnified form black box) demonstrates USPIO within lysosome

USPIOs can be used as a blood pool contrast agent but it is their ability to be taken up by inflammatory cells that has distinguished them [7]. Cellular uptake of USPIOs occurs through a variety of mechanisms. Phagocytosis and receptor-mediated endocytosis are important for uptake of larger particles, whilst smaller particles are internalized by pinocytosis. Although the avidity of macrophage uptake is strongly influenced by particle size and charge, the surface coating is particularly important [8, 9]. As a result of their smaller size, USPIOs are less readily recognized by phagocytic cells and persist in the circulation for longer than other iron particles (plasma half-life 14–30 h in humans) [10, 11]. They are capable of passing through capillary walls, to be taken up by tissue-resident macrophages and neutrophils (Fig. 1) [12–14]. These characteristics allow USPIOs to detect and highlight cellular inflammation within tissues using CMR.

### Imaging methodology

USPIOs induce local magnetic field inhomogeneities that shorten T2 and T2* relaxations times resulting in a signal deficit on magnetic resonance images. USPIOs also have a T1 shortening effect, particularly at low concentrations, and appear bright on T1 weighted images. The T1 shortening effect mainly depends on the strength of the magnetic field, and is higher in lower field strength.

A range of approaches have been used to evaluate USPIO accumulation in tissues. Most simply, images may be qualitatively assessed for signal deficits. However this approach is subjective, and signal deficits due to calcification or other artefacts may be misinterpreted. Manually drawn regions of interest have been used to allow comparison of signal intensity of the target tissue with that of control tissue although discrete focal areas of USPIO accumulation, and thus focal inflammation, may be missed.

Tissue properties, such as the presence of oedema or haemorrhage, can alter image intensities on T2* sequences, and so pre- and post-contrast images need to be compared to delineate the impact of USPIO accumulation. This requires accurate co-registration of these paired scans and adjustments for differences in baseline intensity. A specific region of interest (ROI) map can be drawn and subsequently transferred to each subsequent co-registered image, thus ensuring the signal intensity can be compared for identical sample regions in different scans from the same patient.

**Fig. 2:**
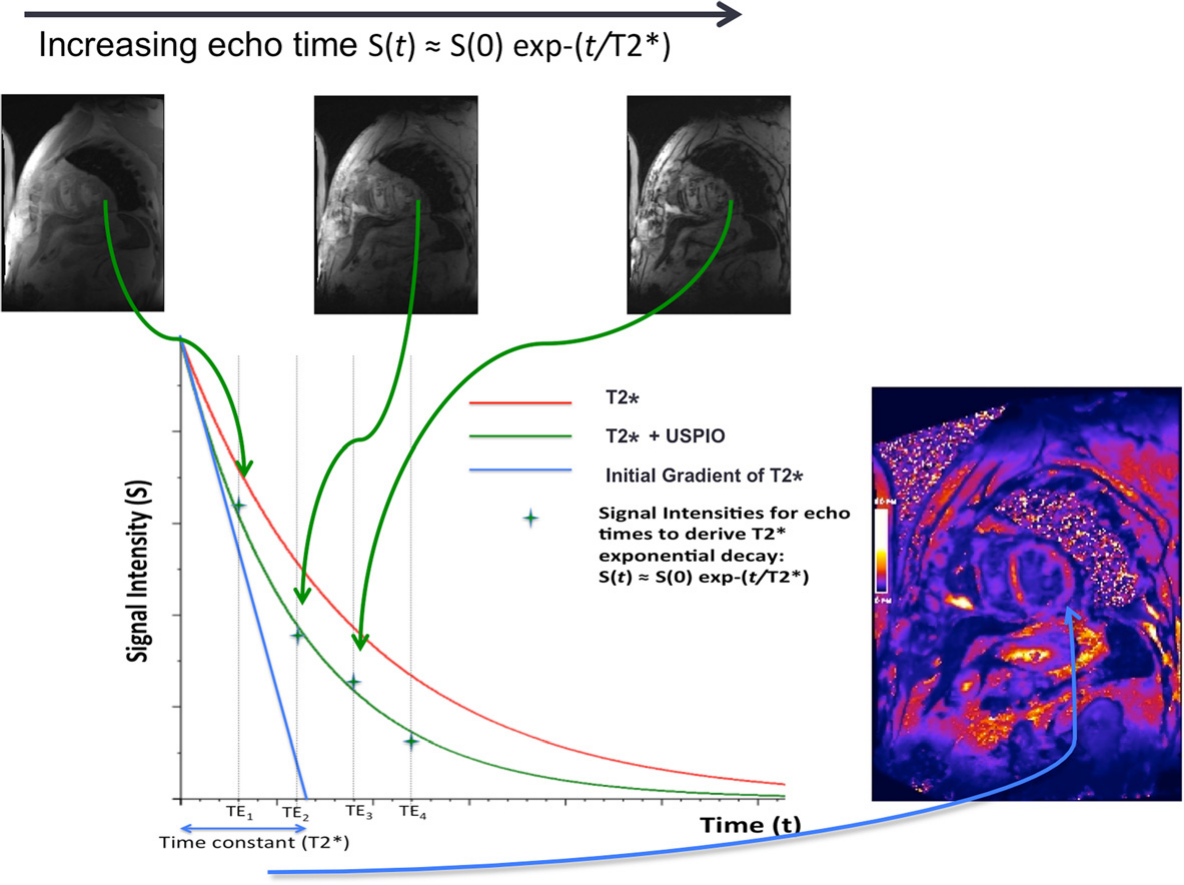
Theoretical T2* exponential decay curves. The T2* curve can be plotted using signal intensities from a region of interest (green crosses) for specific echo times (TEs). In this case, a line of best fit is plotted using the known equation for T2* decay. A T2* map is created from these derived T2* values giving pixel-by-pixel measurements of T2* reported in units of milliseconds, rather than signal intensity of raw images. The red curve describes the decay from pre-USPIO tissue, and the green curved indicated a faster decay due to presence of USPIO. The blue line describes the time constant, T2*

**Fig. 3:**
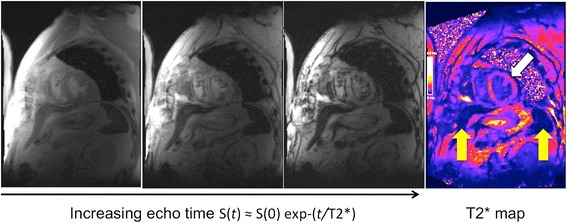
Cardiac T2* Imaging. Multiple images obtained from increasing echo time points (3 time points shown from the left) can be combined to create a T2* map (final image on the right). This map includes the spleen and liver (yellow arrows) and the myocardium (white arrow). These tissues are dark indicating low T2* values consistent with higher USPIO uptake

Rather than assessing focal image brightness at a single echo time, the T2* time constant can be calculated from the exponential decay curve using multiple echo times (Fig. 2). This method provides greater reproducibility, broad applicability throughout the field of view, and independence from T1 effects and a range of imaging variables. In the presence of USPIO, the T2* relaxation rate is increased thus giving a lower T2* value, or higher R2* value (R2* is the inverse of T2*, R2* = 1/T2*). Calculation of these values permits the generation of T2* or R2* maps indicative of USPIO accumulation (Fig. 3).

Various authors have used different techniques to calculate USPIO uptake in tissues, and have reported results using T2, T2* or R2*. This can cause confusion since higher values infer diminished USPIO uptake in T2/T2* weighted images, but higher uptake in R2* maps. For the purposes of this paper, imaging techniques will be described but results reported in terms of “increased USPIO uptake.” In order to account for native R2* values, various authors have used the delta increase in R2* value from successive scans, or factor increase. When pre-USPIO scans have not been performed, it must be assumed that non-inflamed tissue has similar R2* values to pre-USPIO native R2* values.

Finally, it must be noted that USPIO imaging can be affected by artefact. USPIO also shorten T1, and so cause signal enhancement of T1 weighted imaging [15]. However, at high concentration USPIO can cause signal loss with such imaging limiting its use with T1 weighted sequences [16]. The superparamagnetic nature of the particles means that they generate strong local magnetic field inhomogeneities, and it is this magnetic susceptibility that is being imaged by CMR. However this can cause loss of distinction of anatomical borders and distort normal tissues (“blooming artefact”). USPIO will accumulate in the reticulo-endothelial system including the liver and spleen. This accumulation can affect neighbouring structures, and care must be taken not mistake blooming artefact for USPIO uptake.

## Cardiovascular applications

### Atherosclerotic plaque

Given the central role of macrophage biology in the pathogenesis of atherosclerosis, USPIOs have an obvious application in the investigation of atherosclerotic disease. In pre-clinical studies, uptake of USPIOs is demonstrable within numerous atherosclerotic models including aortic plaques of hyperlipidemic rabbits [17, 18] and mice [19] as well as the neointimal hyperplasia following balloon injury [12, 20], and is proportional to plaque macrophage content.

Modulation of inflammation within atherosclerotic plaques can be assessed by USPIO imaging [21]. P38 Mitogen-activated protein kinase (MAPK) is an inflammatory signalling pathway activated by angiotensin II in various vascular cell types [22, 23]. Angiotensin II infusion leads to macrophage accumulation and UPSIO uptake in atherosclerotic plaques of mice [21, 24] that can be inhibited by co-administration of a p38 MAPK pathway inhibitor. Interestingly, this effect was predominantly manifested by a reduction in USPIO uptake by macrophages rather than a reduction in macrophage numbers, suggesting an effect on macrophage activity rather than recruitment. In contrast, the angiotensin II type 1 receptor antagonist, irbesartan, decreased both USPIO uptake and macrophage content in the apolipoprotein E deficient mouse model [25].

USPIO uptake occurs in human carotid atherosclerotic plaques and appears to correlate with the number of iron-laden macrophages on histology [26]. Consistent with the inflammatory cell infiltrate associated with vulnerable plaques, 75 % of ruptured or rupture-prone lesions show USPIO uptake compared to only 7 % of apparently stable lesions. Determining the overall macrophage burden is challenging because of a number of factors. There is a curvilinear relationship between area of signal intensity reduction and USPIO concentration. Signal intensity is also dependent on density of particle accumulation, and a heterogeneous population of macrophages would be expected to have differing degrees of USPIO uptake. The amount of USPIO infused, and by extrapolation perfusion of target tissue, will also determine the magnitude of CMR changes [27].

USPIO uptake and inflammation does not correlate with plaque volume or the degree of luminal stenosis [28], and as already stated USPIO maybe useful in investigating USPIO activity in contrast to concentration [24]. This raises the possibility of using USPIO uptake to monitor disease activity in carotid stenosis rather than using conventional anatomical measurements. For instance, statins reduce inflammation within atherosclerotic plaques as well as systemic markers of inflammation [29, 30] and this has been assessed using USPIO uptake. The ATHEROMA study (Atorvastatin THerapy: Effects on Reduction Of Macrophage Activity) compared the effect of high-dose (80 mg daily) versus low-dose (10 mg daily) atorvastatin on plaque inflammation [31]. Patients underwent UPSIO-enhanced CMR at baseline, 6 weeks and 12 weeks of therapy. Although there were no differences in USPIO uptake over the course of the study in the low-dose group, there was a reduction in USPIO uptake in the high-dose group at both 6 and 12 weeks. This correlated with a reduction in LDL cholesterol and a reduction in micro-emboli count on trans-cranial Doppler [32].

### Abdominal aortic aneurysms

Macrophages are intimately involved in the development, expansion and ultimately rupture of abdominal aortic aneurysms. Preliminary evidence of USPIO uptake in human abdominal aortic aneurysms (AAA) has been described [33, 34]. In a pilot study, we demonstrated that just under half of patients with AAA had focal mural uptake of USPIOs. The aneurysm expansion rate was three-fold higher in patients with USPIO uptake in the aneurysm wall (0.66 *versus* 0.22 cm/year) [35]. Histology of tissue excised at the time of elective surgical repair confirmed co-localization of USPIOs with CD68 immunostaining for macrophages. Thus USPIO-enhanced CMR appears to identify those patients with more rapid disease progression requiring earlier preventative surgical or endovascular intervention to prevent rupture.

### Cerebrovascular disease

Stroke results from an acute disruption to the cerebral blood supply leading to tissue ischemia and eventually necrosis. Inflammatory cells are recruited to the infarct zone, but may extend the injury by interacting with “at risk” cells in the penumbra of the infarct [36, 37]. In a murine model of middle cerebral artery occlusion, USPIO uptake is detected in this penumbra region of infarction [38, 39]. By 7 days the USPIO is confined to the infarct itself, and histology confirms a large population of iron-containing macrophages in the infarcted tissue consistent with migration of macrophages from the penumbra. Further work has indicated that in the setting of established stroke, USPIO leaks through the injured blood–brain barrier accounting for the initial accumulation at the periphery of the infarct and intravascular trapping rather than macrophage uptake [40]. In addition there is widespread uptake resulting from leakage of USPIO into the cerebro-spinal fluid with delivery of nanoparticals to more remote areas. Thus the application of USPIO in such settings is limited although it is possible to track focal USPIO uptake associated with macrophage/microglial infiltration 6 days after cerebral ischaemia, identifying a subacute pathological process [41, 42].

Clinical studies have utilized ferumoxtran-10 in patients 4–5 days after stroke, with imaging at 24–36 h and repeated 48–72 h later [43]. T1- and T2/T2*-weighted imaging reveals parenchymal enhancement that increases between the 2 scans, corresponding to the expected macrophage distribution. These USPIO induced changes do not correspond to conventional gadolinium-enhanced changes, suggesting they occurred independently of blood–brain-barrier breakdown. It could be speculated that these changes may have been due to differences in blood pooling effects due to perfusion changes rather than USPIO inflammatory cell uptake. It would be expected that ischemic volume would correlate with inflammatory burden and CMR changes if USPIOs were being taken up by inflammatory cells. Nighoghossian et al. found no such correlation six days after stroke [44] although the study had a number of limitations including imperfect timing of the scans and the completion of only 5 patients using the more sensitive T2* imaging protocol.

Despite these limitations, a pre-clinical model of the investigation of anti-inflammatory medication in the treatment of stroke has major potential [45]. Using a murine model, the anti-inflammatory agent minocycline can be evaluated after middle cerebral artery occlusion [45]. Minocycline treatment reduced USPIO uptake within the infarct, and was associated with reductions in infarct size, blood–brain barrier permeability and microglia/macrophage counts.

## Future applications

The application of USPIOs to study myocardial inflammation has translational application where the pathology involves substantial monocyte influx into the plaque or tissue [46] (Table 2).

**Table 2:** USPIO in cardiovascular disease

Target	Model & USPIO preparation	Imaging findings
Atherosclerotic plaques	Ferumoxtran-10 imaging of rabbit aorta [12].	UPSIOs identified within aortic atherosclerotic plaques. They are taken up by macrophages.
	Ferumoxtran-10 & ferumoxytol in rabbit aorta [64].	Both USPIO preparations could lidentified within atherosclerotic inflammation. The peak signal for imaging was 2–3 days after ferumoxytol injection, compared to 4–5 days with ferumoxtran-10.
	ApoE^−/−^ mice infused with angiotensin II, or angiotensin II and a p38 MAPK inhibitor with ferumoxtran-10 imaging [21].	The angiotensin II treated animals had the greatest USPIO uptake corresponding with macrophage infiltration. The angiotensin II/p38 MAPK inhibitor group had lower USPIO uptake, which was no different to untreated controls. Modulation of inflammatory cell activity within atherosclerotic plaque could be monitored with USPIO contrast.
	ApoE^−/−^ mice treated with irbesartan were compared to non-treated mice using P904 USPIO [25]. *in vivo* USPIO labelled macrophages compared to *in vitro* USPIO labelling macrophages.	Irbesartan treatment resulted in decreased USPIO uptake compared to controls, which was associated with a significant reduction in macrophage-covered area. The use of *in vitro* labelled macrophages did not produce a significant difference in T2* values despite a difference in macrophage accumulation at histology.
Carotid atherosclerosis	Human carotid plaques using ferumoxtran-10 [65].	USPIOs taken up by macrophages could be identified in human atherosclerotic plaques. High risk plaques took up USPIO more avidly.
	Ferumoxtran-10 uptake within carotid plaques of patients with symptomatic and asymptomatic disease [66].	There was more USPIO signal in “contralateral asymptomatic plaques” compared to “truly asymptomatic” patients. Patients with stroke disease have a higher inflammatory burden within non-culprit carotid artery plaques compared with the plaques from asymptomatic patients.
	Comparison of carotid plaques of patients awaiting CABG to asymptomatic patients using ferumoxtran-10 [67].	Higher USPIO uptake within the CABG group. The plaques of the CABG patients exhibited a USPIO related signal of i 16.4 % compared to 8.4 % in asymptomatic patients. Patients awaiting CABG had higher inflammatory plaque burden.
	The ATHEROMA study (Atorvastatin THerapy: Effects on Reduction Of Macrophage Activity) to investigate the effects of high-dose versus low-dose statin with ferumoxtran-10 imaging [31, 32].	Significant reduction in USPIO uptake in the high-dose atorvastatin group at 6 and 12 weeks. This correlated with favourable reductions in LDL cholesterol and micro-emboli count. Quantitative T2* values showed a highly significant reduction in USPIO-related signal over the course of follow-up. Modulation of plaque inflammation by statins can be monitored by USPIO imaging.
Stroke	Murine model of middle cerebral artery occlusion using ferumoxtran-10 [38].	48 h after stroke, USPIO signal identified within peri-infarct zone. Histology confirmed a large population of iron containing macrophages in the infarcted tissue.
	Murine model with ferumoxtran-10 and T2-weighted imaging with multiple scanning points in the first 72 h after stroke [39].	Disruption of the blood brain barrier leads to leakage of USPIO into the CSF, limiting the specificity of inflammatory cell imaging.
	Spatio-temporal distribution of ferumoxtran-10 was monitored in a rat model of transient cerebral infarction using T1- and T2-weighted CMR sequences [68].	Maximum USPIO related signal occurred on day 2. The technique was successful in achieving non-invasive imaging of inflammation associated with transient ischaemia, but was not sensitive enough to identify increased macrophage infiltration at later time points.
	Murine model to investigate the effects of anti-inflammatory minocycline after middle cerebral artery occlusion using P904 [45].	Treatment reduced infarct size, blood–brain barrier permeability and microglia/macrophage counts. This correlated with decreased R2* value (and USPIO uptake) on imaging as well as tissue iron content.
	Ferumoxtran-10 administered to patients 4–5 days after suffering a stroke with imaging performed 24–36 h and 48–72 h later [43].	T1 weighted imaging revealed parenchymal enhancement that increased between the 2 scans, corresponding to the expected macrophage distribution. T2/T2* weighted imaging revealed increased USPIO enhancement between scans, which the authors interpreted as blood pool effect. These USPIO induced changes did not correspond to conventional gadolinium enhanced changes, suggesting they occurred independent of blood–brain-barrier breakdown.
Myocardial infarction	USPIO agent NC100150 as a blood pool agent in a rodent model of reperfusion after MI [69].	Hyperenhancement of the myocardium by UPSIO was compared to infarct size. USPIO T1-weighted hyper-enhancement was larger than infarction area after reperfusion, but smaller than area at risk. UPSIO corresponded with micro vascular injury and was associated with leakage into the extravascular space.
	Montet-Abou et al. studied fluorescent iron oxide nanoparticles (5–20 nm) in a rodent MI model [70].	Rats with a sham operation and those with MI but not given USPIO did not have significant change in USPIO uptake. The MI group given USPIO had a significant increase in USPIO uptake over the 3 days, with excellent correlation of monocytes/macrophages on histology. CD-68 immuno-staining confirmed co-localisation of fluorescent USPIO particles within macrophages. Rats treated with anti-inflammatory medication showed reduced USPIO signal. This corresponded with less monocyte/macrophage infiltration confirming that USPIO can track inflammation and response to therapeutic intervention within infarcted myocardium.
	Ferumoxytol in human myocardial infarction [46].	USPIO uptake increased significantly in the infarct zone and also in the peri-infarct and remote myocardium to lesser extents.
Cardiac transplant	Synthesised dextran coated USPIO to investigate macrophage accumulation in a rodent cardiac allograft rejection model [71].	Control rodents did not have significant USPIO uptake at baseline. Allograft rodents exhibited large USPIO uptake which was reduced by immunosuppression. Corresponding macrophage counts were greatest in the allograft group and reduced by immunosuppressive treatment indicating that USPIOs can be used to monitor transplant rejection.

### Targeted iron oxide particles

Conjugating iron oxide particles with antibodies allows targeted imaging. Pre-clinical imaging to date has employed 9.4 Tesla CMR. This would be more sensitive in detecting USPIO than clinical CMR systems (1.5 or 3-tesla). In addition, injected unconjugated USPIOs injected directly into the blood stream concentrate within macrophages resulting in high local distribution. It remains to be seen if antibody-labelled USPIOs will be sufficiently concentrated at their target site to allow detection in clinical CMR systems. Specific subsets of monocytes or other cell types could be tracked with successful application of this method. This would allow delineation of the temporal dynamics of cellular and immunological processes by repeated scanning. This has been demonstrated in a pre-clinical model of cerebral ischaemia using USPIOs grafted with a specific peptide targeting vascular cellular adhesion molecule-1 (VCAM-1). This study indicated the potential of VCAM-1 to assess vascular injury.

E-selectin is an adhesion molecule between the endothelium and leukocytes that plays a critical role in the pathogenesis of inflammation [47]. An E-selectin monoclonal antibody-USPIO conjugate has been used to track vascular inflammation in a murine model of contact hypersensitivity [48]. More recently, USPIOs have been conjugated with a scavenger receptor to identify inflammation in atherosclerotic plaques [49].

Another potential confounding factor is that macrophages of different subsets or with different activation status take up USPIO at different rates. This could result in false positive or negative CMR enhancement. Direct labeling of cells with USPIO would avoid this error but published data are limited. Although directly labeling of macrophages with USPIO and delivery through the carotid artery has been successful in producing T2* hypo-enhancement after transient ischaemia, it is associated with increased mortality in a rat model [50].

### USPIO cell labelling and monitoring cell trafficking

The ability to track cells non-invasively *in vivo* would be a valuable technique with a number of potential applications that include inflammatory cell tracking and evaluation of engraftment of cells administered as part of cell-based therapies.

USPIOs can be used to label cells *in vitro* for subsequent *in vivo* tracking. Smooth muscle cells labelled with iron nanoparticles can be imaged when directly injected into either healthy or infarcted myocardium in a pre-clinical model [51]. This technique can be utilized to label human aortic smooth muscle cells incorporated into tissue engineered vascular grafts and implanted into mice [52]. We have also demonstrated that cell tracking can be achieved in vivo in humans using similar approaches with the larger SPIOs [53].

## Summary

USPIOs are taken up by macrophages, and can be identified *in vivo* by CMR scanning. T2 and T2*-weighted scanning provide a sensitive method of assessing USPIO accumulation.

USPIO-enhanced magnetic resonance imaging is a promising method for assessing inflammatory processes associated with a range of cardiovascular diseases including those affecting the atherosclerotic plaque and large arteries. Potential clinical applications are under evaluation and include assessing the effects of novel pharmacological agents and *in vivo* cell tracking to determine the fate of cells administered as part of cell therapy.

## Abbreviations

CABG: Coronary artery bypass grafting
CMR: Cardovascular magnetic resonance
FDG-PET: Fluorodeoxyglucose-positron emission tomography
MAPK: Mitogen-activated protein kinase
MCP-1: Monocyte chemotactic protein-1
MI: Myocardial infarction
RAS: Renin-angiotensin system
ROI: Region of interest
SPIO: Super-paramagnetic iron oxide
TNF-α: Tumour necrosis factor alpha
USPIO: Ultrasmall super-paramagnetic iron oxide
VCAM-1: Vascular cell adhesion molecule 1
